# The Sixteenth International Congress of Medicine

**Published:** 1909-09-11

**Authors:** 


### THE SIXTEENTH INTERNATIONAL CONGRESS OF MEDICINE.

There are few capitals in Europe where an International Congress of Medicine can be held with better effect than Buda-Pest, the capital of Hungary. Within the last twenty-five years a young and virile population has covered the site which it occupies with magnificent buildings and wide streets. The dominant palace and the stately Danube, wider by far than the Thames at Westminster Bridge, and with a current stronger than the outflowing tide, lend an impressiveness to the great city which is all its own. The very slightest touch of orientalism in the people, the buildings, and the trades serves to remind the visitor that only 200 years ago the whole country belonged to the Turks. As G. F. Stevens remarked "The East begins at Buda-Pest." The population, having succeeded beyond all expectation in their building, were determined to show that they were equally great intellectually, and that they could vie, and vie successfully, with the great capitals of the world. It is no slight task to organise a large Congress where every arrangement is submitted to the keen criticism of those who have attended many similar meetings. Buda-Pest is fortunate in possessing two members of the medical profession who are gifted in the highest degree with the powers of tact and arrangement necessary for such a purpose. Professor Müller and Dr. Emil de Grósy, the President and General Secretary of the Congress, must be wholly satisfied with the result of their labours, since it was the unanimous opinion of every nation who attended that the arrangements were as perfect in every particular as it was possible to make them.

The Sixteenth International Congress of Medicine will be remembered less for the scientific work presented at the sections than for the fact that, for the first time, a determined attempt was made to organise the Congress on proper business lines. Originally beginning in small gatherings, the necessary organisation was of the simplest kind—a President and General Secretary with a Treasurer was appointed triennially. These officers being elected for a definite object had no official communication with their predecessors or with their successors, and there was thus no continuity of policy. Honorary officers were not always appointed with discretion, and there was a general feeling that, as the Congress increased in size and the number of members multiplied, the original organisation was no longer sufficient, and new methods must be adopted. Special meetings were held, therefore, and it was determined after prolonged discussion that a permanent committee should be formed with a President, a paid Secretary, and a fixed office. Dr. F. W. Pavy, F.R.S., the eldest and one of the most enthusiastic supporters of International Medical Congresses, was appointed President of the permanent committee. It was decided that the meetings should be held at the Hague, but the nomination of the Secretary and the rate at which he was to be remunerated were left for future deliberation. The committee itself was to consist of not more than fifty members chosen from the various national committees, the President and the Secretaries of these committees being members *ex officio*. There is every hope, therefore, that the affairs of the Congress will prosper under this arrangement, and that an unwieldy growth which was becoming troublesome will be brought again into proper proportions by judicious lopping.

The sectional meetings were held in the Polytechnic, in the old Parliament House, and in the lecture theatre attached to the National Museum. They were well attended, but nothing of any great or novel interest was brought forward. In the Surgical Section the subjects of appendicitis and of the means by which asepsis could be secured before operation received special attention, whilst the consideration of serum diagnosis and immunity in the Medical Section appeared to have taken the place of the older clinical papers. Indeed, immunity in some of its many forms may be said to have pervaded every section of the Congress, and it is apparent that this important subject is now enjoying the thoughts of the best minds throughout the world. The results at present are contradictory, apparently because they are veiled by a series of working hypotheses, each with a curious jargon of its own. A few salient points stand out already, and there can be little doubt that within a few years the subject will be greatly simplified and valuable results will be obtained prophylactically as well as in the actual cure of disease.

The subjects of the general addresses had been carefully selected; they were well delivered, and the attendance in each case was crowded. Dr. Holländer lectured upon Disease as depicted by Art in America before the arrival of Columbus, and showed by means of lantern slides the deformities produced by syphilis and lupus as they had appeared to the prehistoric potters. Dr. Bashford delivered a useful and interesting address on Cancer, in which he gave a clear statement of the present position of the question of its origin as a result of the work of the Imperial Cancer Research Laboratories. Dr. Loeb lectured upon Artificial Parthenogenesis and its bearing upon the Physiology and Pathology of the cell, and Mons. Laveran, of Paris, spoke upon Exotic Pathology.

The entertainments were well planned, and a special ladies' committee, with Madame Bokai at its head, issued a separate programme, and saw that it was carried out to the satisfaction of all who took part in it. For ladies and gentlemen alike there was a soirée of welcome at the Gallery of Fine Arts in the City Park on the first night; a soirée at the municipal buildings on the second day, and a ladies' evening on Tuesday. The Archduke Joseph received the members of the Congress at the Palace on Wednesday, and graciously spoke to a large number of the official delegates who had the honour of being presented to him by H.E. Count Albert Apponyi, the Minister of Public Instruction. Last, and perhaps best of all where all was good, was the soirée given by Count Albert

Apponyi at the Park Club, the most select in Hungary and the most beautifully housed. There was a gala performance at the theatre on the Wednesday, to compensate the ladies who were not invited to the Court reception.

It was decided at the last meeting that, upon the invitation of Sir Edward Grey, Secretary of State for Foreign Affairs, the seventeenth International Congress of Medicine should hold its sittings at some town in Great Britain or Ireland in the course of the year 1913. Great efforts must be made by the whole medical profession if this meeting is to eclipse, or even to equal, the one which has just been held. Better papers may be presented, but it will be extremely difficult to equal the politeness and the organisation which have been so conspicuous at the Buda-Pest meeting.

The Hungarians did wisely to extend an invitation to the medical profession and to hold this meeting in the capital. Buda and its neighbourhood contains many natural springs whose waters are known and used throughout the world. Personal inspection of the sources has quickened the interest of a large number of medical men in the waters, and has shown how natural they are and how carefully they are preserved from contamination. But the Congress has done more than this. It has shown to all who attended it a great nation in the making—a nation united by a language difficult to acquire and yet of sufficient flexibility to render idiomatically the ideas of Herbert Spencer, of Conan Doyle; a nation of high ideals and filled at present with boundless enthusiasm. All promises well now, but it remains to be seen whether the nation as a whole possesses those powers of solid industry and dogged perseverance which have brought the Anglo-Saxons to the front, or whether the fatal inheritance of a long Oriental despotism, coupled with a fertile soil and a beautiful climate, will not prove a bar to the continued and strenuous exertion which alone can achieve the great purposes held in view by the present generation. Much still remains to be done. Kra von Krafft-Ebing's work is a little too commonly displayed in the shop-windows, there is a double monetary system in common use, and with a magnificent river flowing through the town rowing, as a sport taken seriously, is non-existent. So much has been done, on the other hand, within a marvellously short space of time, that the future looks almost assured, and if the authorities rule as well and as wisely during the next two or three generations as they have done in the past Hungary should become a great nation of which England may be proud, for she is modelling her constitution upon that of Great Britain. In this endeavour we heartily wish her success.

